# Developmental Profile and Sexually Dimorphic Expression of Kiss1 and Kiss1r in the Fetal Mouse Brain

**DOI:** 10.3389/fendo.2013.00140

**Published:** 2013-10-11

**Authors:** John Gabriel Knoll, Colin M. Clay, Gerrit J. Bouma, Timothy R. Henion, Gerald A. Schwarting, Robert P. Millar, Stuart A. Tobet

**Affiliations:** ^1^Oregon Health and Science University, Portland, OR, USA; ^2^Biomedical Science, Colorado State University, Fort Collins, CO, USA; ^3^Cell Biology, University of Massachusetts Medical School, Worcester, MA, USA; ^4^MRC Receptor Biology Unit, University of Cape Town, Cape Town, South Africa; ^5^Centre for Integrative Physiology, University of Edinburgh, Edinburgh, UK; ^6^Mammal Research Institute, University of Pretoria, Pretoria, South Africa; ^7^Biomedical Science and Biomedical Engineering, Colorado State University, Fort Collins, CO, USA

**Keywords:** kisspeptin, KISS1R, arcuate nucleus, RP3V

## Abstract

The hypothalamic-pituitary-gonadal axis (HPG) is a complex neuroendocrine circuit involving multiple levels of regulation. Kisspeptin neurons play essential roles in controlling the HPG axis from the perspectives of puberty onset, oscillations of gonadotropin releasing hormone (GnRH) neuron activity, and the pre-ovulatory LH surge. The current studies focus on the expression of kisspeptin during murine fetal development using *in situ* hybridization (ISH), quantitative reverse transcription real-time PCR (QPCR), and immunocytochemistry. Expression of mRNA coding for kisspeptin (KISS1) and its receptor KISS1R was observed at embryonic (E) day 13 by ISH. At E13 and other later ages examined, Kiss1 signal in individual cells within the arcuate nucleus (ARC) appeared stronger in females than males. ISH examination of agonadal steroidogenic factor-1 (*Sf1*) knockout mice revealed that E17 XY knockouts (KO) resembled wild-type (WT) XX females. These findings raise the possibility that gonadal hormones modulate the expression of Kiss1 in the ARC prior to birth. The sex and genotype differences were tested quantitatively by QPCR experiments in dissected hypothalami from mice at E17 and adulthood. Females had significantly more Kiss1 than males at both ages, even though the number of cells detected by ISH was similar. In addition, QPCR revealed a significant difference in the amount of Kiss1 mRNA in *Sf1* mice with WT XY mice expressing less than XY KO and XX mice of both genotypes. The detection of immunoreactive KISS1 in perikarya of the ARC at E17 indicates that early mRNA is translated to peptide. The functional significance of this early expression of *Kiss1* awaits elucidation.

## Introduction

The hypothalamic-pituitary-gonadal (HPG) axis is a complex neuroendocrine circuit responsible for controlling physiological and behavioral reproductive function. The classical description of the circuit begins with gonadotropin releasing hormone (GnRH) neurons, which reside in the basal forebrain/hypothalamus, secreting GnRH into the hypophyseal portal vasculature where it is transported to gonadotropes in the anterior pituitary (1, 2). At least one other cell type is now described as a regulatory component of this circuitry (3). Cells that express the metastasis suppressor gene *Kiss1*, whose protein product is commonly known as kisspeptin (KISS1), reside in and around two specific locations in the hypothalamus; the rostral periventricular portion of the pre-optic area (POA; sometimes referred to as the RP3V for the rostral periventricular area of the third ventricle), and the caudal region in and around the arcuate nucleus (ARC) (4, 5). KISS1 stimulates GnRH neuron activity and *Kiss1*/KISS1 expression and release is regulated by circulating gonadal hormones (6, 7). All evidence to date indicates that cells that synthesize KISS1 are functionally upstream of GnRH neurons.

Much effort has gone into characterizing the role played by KISS1 in the HPG axis. Work with targeted disruption of the genes encoding KISS1 or its cognate receptor KISS1R has shown that KISS1/KISS1R is essential for puberty and adult reproductive competence (8–10). Two populations of kisspeptin neurons have been identified. One population of neuronal cell bodies residing in the RP3V is responsible for mediating the pre-ovulatory GnRH/LH surge and positive feedback actions of estradiol, while the more caudal population around the ARC is responsible for controlling the oscillatory GnRH pulses and negative feedback actions of estradiol (11–13).

Data has revealed that in pre-pubertal and adult animals *Kiss1* expression is regulated by estradiol and testosterone (7). Interestingly, the regulation of *Kiss1* is opposite in the different regions that contain *Kiss1* neurons. In the RP3V, steroid feedback is positive and up-regulates *Kiss1* and in the ARC steroid feedback is negative and decreases *Kiss1* (14). Several approaches involving genetic modifications have been used to study hormonal influences on *Kiss1*, including mutations that preclude GnRH synthesis (15), and those that preclude estradiol synthesis (16). The current study takes advantage of transgenic mice in which the steroidogenic factor-1 (*Sf1*) gene was disrupted. These Sf1 knockouts (KO) mice do not develop fetal gonads and adrenal glands, and as such there is no endogenous fetal sex-steroid production (17). This enables us to determine if *Kiss1* levels are regulated by sex steroid in the fetal brain. Our hypothesis is that if gonadal steroids regulate fetal ARC *Kiss1*, *Sf1* disrupted (KO) XY mice (that develop as phenotypic females) should have more *Kiss1* than wild-type (WT) XY, and display expression levels similar to XX mice.

The goal of the current research was to determine the expression pattern of *Kiss1* during fetal development and pre-pubertally, as well as to assess *Kiss1* levels in fetal agonadal mice. To accomplish this, both *in situ* hybridization (ISH) using digoxigenin (DIG)-labeled riboprobes and real-time PCR quantitative reverse transcription real-time PCR (QPCR) were used as complementary approaches to assess regional localization and expression levels, respectively, in brain tissue collected from embryonic day 13 (E13) to post-natal day 35 (P35). Expression of *Kiss1* and *Kiss1r* was detected at E13 and maintained through puberty. Immunohistochemistry (IHC) in fetal and adult tissue revealed a KISS1 expression pattern similar to that of *Kiss1*, indicating mRNA is translated into biologically active protein in early development. A sex difference in *Kiss1* expression was seen at specific fetal, post-natal, and peri-pubertal ages, and levels in females always showed significantly higher mRNA levels than males. Examining *Kiss1* in fetal brain tissue collected from agonadal *Sf1* KO mice, revealed *Kiss1* mRNA expression in XY KO mice resembled WT XX more than WT XY mice, supporting the hypothesis that the differentiated gonad regulates sexual dimorphism in *Kiss1* expression even prior to birth.

## Materials and Methods

### Animals

GnRH-eGFP (17) and *Sf1* KO (18) mice were housed in the Painter Center of Laboratory Animal Resources at Colorado State University and maintained in plastic cages with aspen bedding (autoclaved Sani Chips, Harlan Teklad, Madison, WI, USA) and environmental enrichments on a 14:10 h light:dark cycle with free access to food (#8640, Harlan Teklad, Madison, WI, USA) and tap water. Animal care and handling was in accordance with the Colorado State University Animal Care and Use Committee guidelines.

For fetal tissue collections, female and male mice were housed together overnight, and the following day considered to be embryonic day 0 (E0) if vaginal plugs were observed. At the appropriate age, pregnant dams were deeply anesthetized with ketamine/xylazine (80 and 8 mg/kg, respectively), embryos were removed individually by Cesarean section, and a crown-rump-length measurement was taken to verify gestational age (approximately 10 mm at E13, 14 mm at E15, and 19 mm at E17) (19). GnRH-eGFP mice were homozygous for eGFP (CBB6/F1 background), and heterozygous *Sf-1* mice (C57BL/6 background) were bred to produce WT and KO embryos. Tissue from each fetus was collected prior to perfusion for genotyping PCR using primers for *Sry* to determine genetic sex, and primers for *Sf1* to identify *Sf1* KO animals (20). After removal, each pup was perfused transcardially with 2 ml 4% paraformaldehyde in 0.1 M phosphate buffer (PB; pH = 7.4) using a hand held 10 ml syringe. Heads were post-fixed overnight in fresh perfusion fixative then stored at 4°C in 0.1 M PB prior to sectioning.

For post-natal tissues, post-natal day (P) 12 and P35 animals were anesthetized with ketamine/xylazine (80 and 8 mg/kg, respectively) and perfused with 10 ml Heparin/Saline followed by 10 ml 4% paraformaldehyde. Heads/brains were post-fixed overnight as above and stored in 0.1 M PB prior to sectioning.

### Probes for *in situ* hybridization

The templates for generating DIG-labeled riboprobes to *Kiss1* and *Kiss1r* were obtained from Invitrogen Corporation as cDNAs in the vector pT7T3-Pac, and prepared using DIG RNA labeling mix (Roche Applied Sciences #11277073910) according to manufacturer’s instructions. Antisense riboprobes to *Kiss1* were transcribed from an *Eco*RI-linearized cDNA (GenBank accession #AA023588) that includes the entire kisspeptin pre-protein coding sequence (base pairs 38–416 of reference sequence NM_178260). *Kiss1r* antisense riboprobes were generated from *Eco*RI-linearized cDNA (accession #BF470621) complementary to base pairs 1716–2842 of *Kiss1r* reference sequence NM_053244), representing nearly the entire *Kiss1r* coding sequence with the exception of 234 base pairs within alternatively spliced exon 4. Sense probes were generated from the same plasmids linearized with *Not*I. No reaction product was observed in sections incubated with sense probes using ISH.

### *In situ* hybridization

*In situ* hybridization was performed as described previously (21) using free-floating tissue sections. Brains were embedded in 5% agarose containing 1% diethyl pyrocarbonate (DEPC; Sigma, D5758) and sectioned at 100 μm using a vibrating microtome (Leica VT1000s) and collected in 0.05 M phosphate buffered saline (PBS) containing 1% DEPC. All sections from fetal brains were collected in one mesh-bottomed container (referred to as a boat) while sections from post-natal brains were alternated between two boats. Sections were then treated with 6% hydrogen peroxide in 0.05 M PB containing 1% Tween-20 and 1% DEPC (PBT) for 1 h followed by three 5 min washes in PBT. After washing, sections were treated with 10 μg/ml proteinase K (Sigma, P2308) in PBT for 15 min, 10 min in 2 mg/ml glycine in PBT, and two 5 min washes in PBT. Next, sections were post-fixed in 4% paraformaldehyde/0.2% glutaraldehyde in PBT for 20 min and washed twice for 5 min in PBT. All incubations and washes up to this point were done on a shaker at room temperature. Following post-fixing, sections were transferred to scintillation vials containing 2 ml of 50% formamide in saline-sodium citrate (SSC; Sigma, S6639) buffer containing 8.8 U/ml heparin (Sigma, H9399), 0.05 mg/ml yeast tRNA (Sigma, R5636), and 0.024 mg/ml dextran sulfate (Sigma, D8906) (collectively pre-hybridization solution) for 1 h shaking at 60°C. DIG labeled riboprobes (1 μg/ml) were denatured by incubating for 5 min in an 85°C water bath and added to the vials containing the sections. Probes were hybridized overnight on a shaker at 60°C.

The following day, sections were transferred back into boats and washed three times for 30 min in a 50% formamide/25% SSC solution, then three times for 30 min in 50% formamide/10% SSC (all while shaking at 60°C). Sections were then washed three times for 5 min in Tris buffered saline (TBS) containing 1% Tween-20 (TBST) then pre-blocked in 10% sheep serum in TBST for 1 h (shaking at room temperature). Sections were then incubated overnight (while shaking at 4°C) in a solution of 1% sheep serum in TBST containing alkaline phosphatase conjugated anti-DIG antibody (Roche, 11093274910) at a concentration of 1:2000.

On the third day, sections were washed three times for 5 min, then five times for 1 h in TBST on a shaker at room temperature, and incubated overnight at 4°C in TBST. On the final day, sections were washed at room temperature three times for 15 min in a solution of 0.1 M NaCl, 0.1 M Tris (pH = 9.5), 0.05 M MgCl_2_, and 1% Tween-20 (collectively NTMT). Sections were then incubated on a shaker in the dark at room temperature in a solution containing 10% polyvinyl alcohol, 1 mM Levamisol (Vector Laboratories, Burlingame, CA SP5000, USA), 2% NBT/BCIP (Roche, 11681451001), 0.1 M Tris (pH = 9.5), 0.1 M NaCl, and 0.005 M MgCl_2_ (color detection mix). Sections were left in the color detection mix until dark purple reaction product was visible under a dissecting microscope, at which point the reaction was terminated for all tissues of the same age. To stop the color reaction, sections were washed twice for 5 min in NTMT in the dark, incubated in 0.1 M TBS containing 0.001 M EDTA for 30 min, and finally twice for 10 min in PBT (on a shaker at room temperature). The procedures were designed to process all tissues at the same time to facilitate qualitative comparisons between different genotypes.

### Immunohistochemistry

Immunocytochemical procedures were standard and performed as described previously (22). Briefly, 50 μm serial sections from 4% paraformaldehyde perfusion-fixed peri-pubertal (P35) or fetal (E17) brains were cut using a vibrating microtome (Leica VT1000s) and collected into mesh-bottomed boats. Sections were pretreated with 0.1 M glycine then 0.5% sodium borohydride in 0.05 M PBS. Sections were blocked with 5% normal goat serum (NGS) and 1% hydrogen peroxide in 0.05 M PBS. Sections were incubated with rabbit derived KISS1 antibody directed against rodent kisspeptin-10 (provided by Dr. Alain Caraty) at a final dilution of 1:10,000 for two nights (23). Primary antibody was localized with a biotinylated donkey anti-rabbit secondary antibody (Jackson Immunoresearch, 711-065-152) used at a concentration of 1:2500 for 2 h. Signal was amplified using the Vectastain ABC Elite reagents (Vector Laboratories, PK6100) at a concentration of 1:330 for 1 h. Finally, the reaction was visualized using diaminobenzidine (DAB; 0.025%)/nickel ammonium sulfate (0.2%) to produce a dark purple/black reaction product. Although it has been reported that the antiserum does not cross react with related RF-amide peptides such as RFRP-1 and RFPR-3 (9, 10) this was not fully verified as some cells were detected in regions such as the dorsomedial hypothalamus where no cells were detected by ISH (Data not shown). Specificity of the IHC was established by omission of the primary antibody.

### Dual-label ISH/IHC

Dual-label ISH/IHC experiments were carried out exactly as single-label experiments with the exception that the two protocols were run in series. In this case, ISH for *Kiss1r* was performed first, followed immediately by IHC for GnRH. Primary antibody for these experiments was rabbit-anti-GnRH (Affinity Bioreagents #PA1-121) used at a concentration of 1:200. For visualization, the IHC was developed using Cy3-conjugated streptavidin (Jackson Immunoresearch #016-160-084) at a concentration of 1:500.

### Quantitative reverse transcription real-time PCR

Tissue was collected fresh from fetuses (E17) and adults ( ∼P50) and flash frozen on dry ice then stored at −80°C until homogenized for RNA isolation. Brains were removed from GnRH-eGFP and Sf1 KO mice in ice cold DEPC PBS. Genetic sex was confirmed by PCR for the presence of *Sry*. Pregnant mice were anesthetized with a mixture of Ketamine and Xylazine (80 and 8 mg/kg respectively) and embryos were removed individually by Caesarian section. Following brain removal, a hypothalamic block was dissected (caudal to the optic chiasm and rostral to the mammillary body) and a small ( <10 mg) wedge of the mediobasal hypothalamus was collected for analysis by making a bilateral triangular excision starting at the middle of the third ventricle and extending to the lateral boundary of the hypothalamus. Adult mice were anesthetized with a mixture of Ketamine and Xylazine (80 and 8 mg/kg respectively) and the brains were dissected out, blocked in the coronal plane rostral to the optic chiasm and caudal to the mammillary bodies, then cut coronally midway between the optic chiasm and the median eminence (ME) to divide the block into rostral and caudal portions. For the rostral portion containing the periventricular portion of the pre-optic area/anterior hypothalamus, a bilateral triangular excision was made extending from the center of the anterior commissure to the basolateral boundary of the hypothalamus. For the caudal portion containing the ARC, an excision similar to that made for the embryos was collected. At both ages cortical pieces were taken for negative controls in the QPCR assays. All dissections were performed using a stereo dissecting microscope.

RNA was isolated using the Qiagen RNeasy Mini Kit (Qiagen Sciences, #74104). Briefly, excised brain pieces were pulverized using a motorized tissue grinder and RNase free tubes and disposable pestles (VWR cat. #47747-370 and #47747-366), then homogenized in the supplied homogenization buffer and procedures were followed according to manufacturer’s instructions. Once eluted, the RNA concentration and purity of each sample was determined by spectrophotometry and samples were stored at −80°C until reverse transcription (RT).

For GnRH-eGFP mice, RT was performed using the Thermo Verso RT kit (Fisher cat #AB1453B) using random hexamer primers for first strand synthesis according to manufacturer’s instructions. For *Sf1* KO mice, RT was performed using the qScript cDNA Synthesis kit (Quanta Biosciences cat #95047) which contains a mixture of random hexamers and oligo(dT). To insure uniformity between samples, all RNA isolates were diluted to 100 ng/μl of which 5 μl (500 ng total RNA) was added to each 20 μl reaction. RT was performed less than 24 h prior to QPCR to minimize potential sample degradation.

For GnRH-eGFP animals, QPCR was performed using HotStart-IT SYBR Green QPCR Master Mix (2X; USB Corporation, Cleveland, OH 75762, USA). According to the HotStart-IT protocol, 20 μl reactions were run of with 2 μl cDNA. For *Sf1* KO animals, QPCR was run using Light Cycler 480 SYBR Green I Master Mix kit (Roche cat #04-707-516001) using 4 μl cDNA in each 20 μl reaction. All QPCR experiments were performed in a LightCycler 480 system (Roche Applied Sciences). Cycling parameters were as follows: hot start at 95°C for 1 min; 45 cycles of amplification/quantification at 95°C for 10 s, 60°C for 30 s, and 72°C for 30 s during which time fluorescence was measured. Melting curve analysis was performed using continuous fluorescence acquisition from 65–97°C. These cycling parameters generated single amplicons for both primer sets used according to the presence of a single melt peak. Primers were designed to amplify intron spanning regions of *Kiss1* and *Rn18s* (reference gene) to investigate the gene of interest and monitor amplification efficiency. The two sets of primers used for this experiment were as follows: *Kiss1* forward 5′-TGATCTCAATGGCTTCTTGGCAGC-3′ and reverse 5′-CTCTCTGCATACCGCGATTC CTTT-3′; *Rn18s* forward 5′-AGGGGAGAGCGGGTAAGAGA-3′ and reverse 5′-GGACAGGACTA GGCGGAACA-3′.

For absolute quantification of kisspeptin transcript, the same plasmid used to generate the *Kiss1* probe was used as a standard. Since each plasmid contains a single copy of the *Kiss1* gene, and using an approximation of the total molecular weight of the plasmid ( ∼3.7E−12 μg/copy) the concentration of plasmid was calculated in terms of number of copies per μl of solution. A 10-fold serial dilution series was used to generate a standard curve ranging from ∼1E^11^ copies/μl (0.4 μg/μl) to ∼100 copies/μl. This dilution series was run in the same plate as each experiment and was used to generate a standard curve from which the concentration (in number of copies/well) was calculated for each sample. This number was then used to calculate the number of copies of *Kiss1* in E17 and adult male and female brains [copies/well ÷ (microliter cDNA/well) = copies/microliter cDNA ÷ (microliter RNA/20 μl cDNA) = copies/microliter RNA × Dilution Factor = copies/microliter RNA (undiluted) × Total RNA Volume = Total Copies/dissection].

To control for contamination, several controls were used. First, pieces of cortex not expected to express *Kiss1* were collected from each brain at the time of tissue collection. These tissues were treated the same as all other tissue pieces and did not generate a signal above background during QPCR. During the RT, two additional controls were used; one reaction with no RNA, and one with RNA (from an adult female rostral POA) but no reverse transcriptase. Controls from each of the previous steps were run through all subsequent steps. Real-time PCR controls included all of the previous controls and one reaction containing no cDNA (water control). None of the negative controls produced a signal above background in QPCR (data not shown). Finally, standards and samples were run in triplicate to verify accuracy; calculated crossing point variance was below 2% for replicated samples.

### Statistical analysis

For each age group in each experiment three animals of each sex and genotype from a minimum of two different litters were used for quantitative analysis. To determine the number of cells expressing kisspeptin, all cells (with obvious nuclear voids) with visible reaction product in all sections from a given brain were manually counted at high magnification by an investigator blind to the sex of each animal. For fetal brains this meant counting all sections from the entire brain (all sections collected in a single boat) while for older animals it meant counting all sections from half of the brain (sections were alternated between two boats). The reason for collecting all sections from the embryonic brains was to ensure that important sections were not lost due to the smaller size of the brains. For comparison purposes, the total number of cells counted for each embryonic brain was divided in half to generate the equivalent of the number of cells per half-brain. This method was validated by alternating sections from a subset of fetal brains into two boats and counting the number of cells per half-brain; the resultant average was approximately equal to the average of half the total cells per whole brain.

For QPCR, the crossing point was determined according to the absolute quantification/second derivative maximum method in the LightCycler 480 SW 1.5 software. From this number, the total copy number was calculated based upon the previously described standard curve. To verify the results of the standard curve absolute quantification method, relative expression analysis was performed by normalizing *Kiss1* to *Rn18s* signal. Both methods generated similar results and the absolute quantification method results were selected for presentation. Cycling parameters were set up such that the exponential amplification phase had been reached for all positive samples (45 cycles total). Any sample that did not exhibit an appropriate amplification curve, a single melt curve, or that came up after 40 cycles was assumed to be a false signal and was not analyzed. Negative control samples were never observed to generate a signal before 40 cycles.

Statistical analysis was performed using SPSS 13.0 or JMP 10.0 for Mac OSX. For cell count analysis, the total number of cells per half-brain and the sex of the animal were analyzed by two-way ANOVA for sex and age. For QPCR data from *Sf-1* embryos two-way ANOVA for sex and genotype was used, with Least Square Means Student’s *t* as a *post hoc* test. For adult animals, even though there were two regions per animal, due to the greater variance in the rostral/POA region compared to the caudal/ARC region, one-way ANOVA was conducted on the rostral region separately from a two-way analysis in the caudal/ARC region.

## Results

### *Kiss1*/KISS1 and *Kiss1r* regional localization in the fetal brain

**Figure 1:**
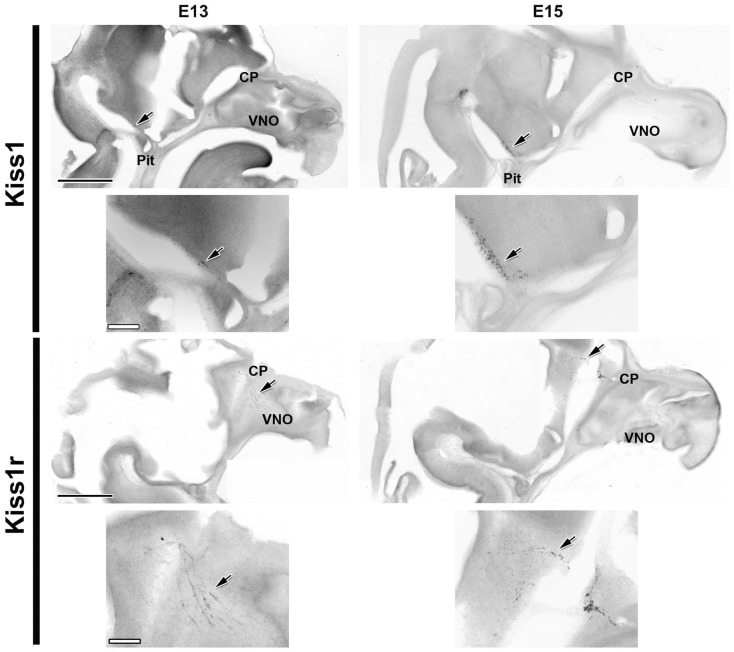
***Kiss1* and *Kiss1r* are expressed as early as E13**. Developmental changes in the expression of *Kiss1* (top four images) and *Kiss1r* (bottom four images) are shown in digital images from sagittal sections of female mice at E13 (left) and E15 (right). Smaller images are higher magnifications of the same sections displayed immediately above them. Note the change in location of the *Kiss1r* positive cells from the nasal compartment to the forebrain between E13 and E15 reminiscent of the migrating GnRH neuron population. Arrows indicate the location of mRNA positive cells. CP, cribriform plate; Pit, pituitary; VNO, vomeronasal organ. Scale Bars: black = 1 mm, white = 200 μm.

*Kiss1* was detected by ISH at E13 in a few cells (arrows in Figure 1 left) near the ME. Two days later (E15) the total number of cells expressing *Kiss1* had greatly increased (from approximately 30 cells/half-brain to approximately 400 cells/half-brain), although the distribution remained similar (Figure 1). Cells containing *Kiss1* were found in approximately eight sequential sections, representing a distance of greater than 800 μm in all ages examined.

Expression of *Kiss1r* was detected at E13 in both sexes by ISH. At this age, the only cells containing *Kiss1r* were found in the nasal compartment in a pattern similar to the pattern of migrating GnRH neurons. By E15 a large proportion of this population of cells had apparently migrated to and across the cribriform plate into the ventral forebrain (Figure 1), again suggestive of migrating GnRH neurons. With each successive age, the pattern of *Kiss1r* expression matched the typical distribution of GnRH neurons, with older animals having a high density of *Kiss1r* expression around the diagonal band of Broca (DBB) and surrounding the organum vasculosum of the lamina terminalis (OVLT) as well as a more dispersed expression as far caudal as the optic chiasm. In addition, *Kiss1r* containing cells were seen in a highly restricted population of cells in the habenula as early as E17 (data not shown). No sex differences were observed in *Kiss1r* expressing cells at any age or in any region (data not shown).

**Figure 2:**
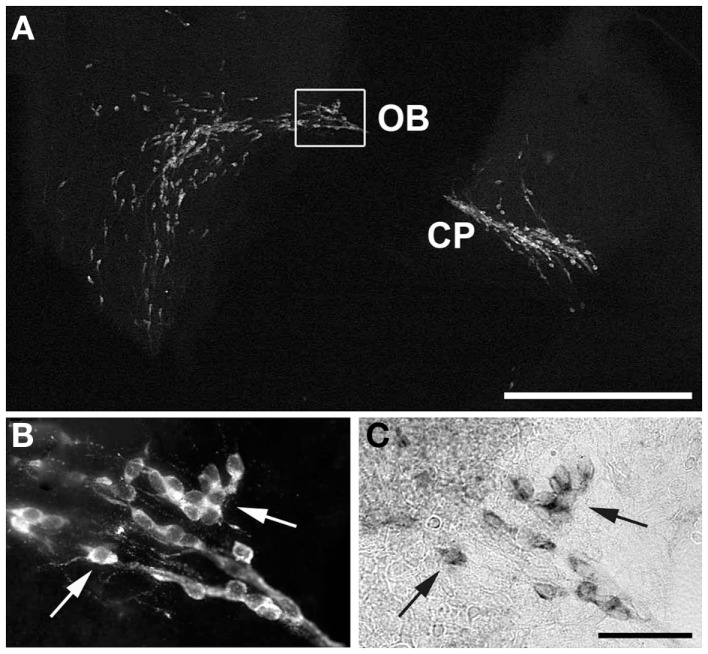
**Co-localization of GnRH and *Kiss1r* in migrating neurons**. *Kiss1r* and GnRH were co-localized in migrating GnRH neurons in E15 mice. **(A)** shows a digital image of a fluorescent IHC for GnRH in a sagittal section from an E15 female also positive for *Kiss1r*. **(B)** (GnRH) and **(C)** (*Kiss1r*) show higher magnification images of the boxed area in **(A)**, with arrows to indicate co-expressing cells. Compare the GnRH pattern in A to the *Kiss1r* pattern seen in the bottom right corner of Figure 1. CP, cribriform plate; OB, olfactory bulb. Scale Bars: white = 500 μm, black = 100 μm.

To verify that the cells seen to express *Kiss1r* in previous experiments were GnRH neurons, dual-label ISH/IHC were performed on E15 (Figure 2). As illustrated, GnRH immunoreactivity was seen in a pattern corresponding to that of the *Kiss1r* expression pattern at the same age (compare with Figure 1). High magnification images of fluorescently labeled GnRH (B) and DIG labeled *Kiss1r* (C) shows that, in fact, cells expressing *Kiss1r* at E15 were migrating GnRH neurons.

**Figure 3:**
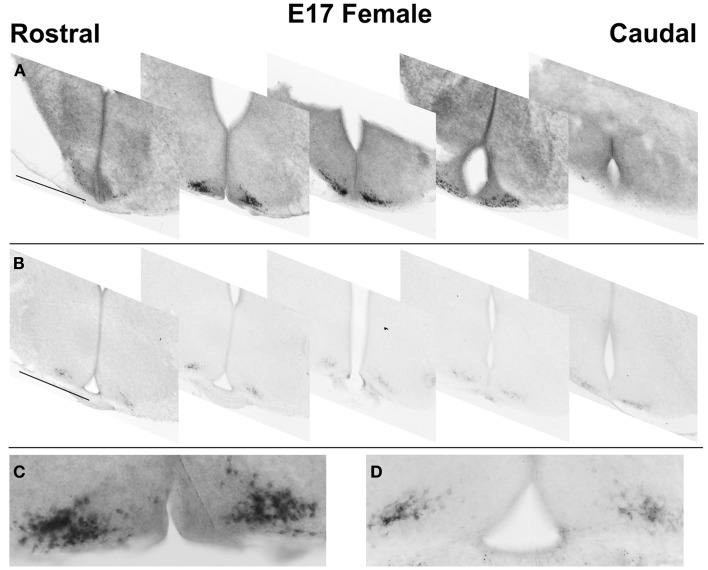
**Embryonic distribution of *Kiss1/*KISS1 expressing cells**. Digital images of representative sections showing the rostral-caudal distribution of cells expressing *Kiss1* **(A)** and kisspeptin-10 immunoreactivity **(B)** in the ARC. Expression extends over 800–1000 μm. High magnification digital montages, taken from coronal plane 2 in **(A,B)**, highlight differences in cell detail such as nuclear voids in ISH reaction product **(C)**, but not IHC **(D)**. Scale bars: **(A,B)** = 500 μm; **(C,D)** = 250 μm.

To determine if *Kiss1* is translated into processed KISS1 peptide during development, serial sections from fetal (E17) brains were examined by IHC. Immunoreactive kisspeptin (KISS1; Figures 3B,D) was detected using an antiserum raised against the biologically active decapeptide. A similar distribution to that seen using ISH was observed (Figures 3A,C). In fetal mice, KISS1 was only found in a distribution of cells in the ARC, and cell bodies were clearly identifiable in fetal ARC.

### Relative differences in Kiss1 localization patterns in mouse fetal and early post-natal brain

**Figure 4:**
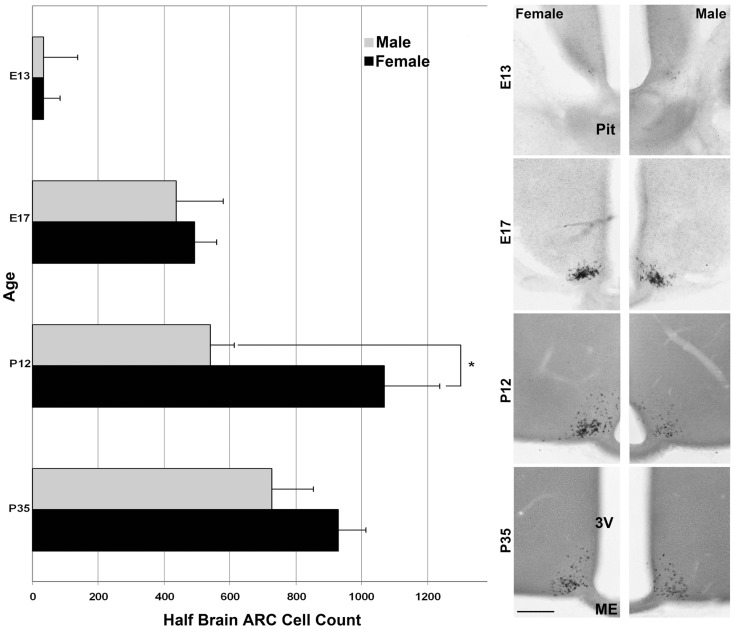
**Developmental changes in cells expressing *Kiss1* in the region of the ARC**. The number of cells expressing *Kiss1* in and around the ARC differs with age (*n* = 3 per group) shown in coronal sections. A transient sex difference in the number of ARC cells was evident at P12 (mean ± SEM; *p < 0.01). Note the difference in the optical density of reaction product between males (images in the right column) and females (left column). This difference is most visibly evident at P12 and P35 but is notable at all ages examined at higher magnification. Pit, pituitary; 3V, third ventricle, ME, median eminence. Scale bar = 100 μm.

For the ages of E15 (Figure 2) and E17 (Figure 4) the pattern of cells expressing *Kiss1* remained unchanged. In addition, there appeared no sex difference in the number of cells expressing *Kiss1* (E15 not shown; E17, Figure 4). At P12, the number of cells expressing *Kiss1* was dramatically higher in the female compared to the male. This is the only age examined at which there was a significant sex difference in the detectable number of cells expressing *Kiss1* in and around the ARC [Figure 4; *F*(1,4) = 8.2, *p* < 0.05]. There was no sex difference in the number of cells expressing *Kiss1* at E13, E15, E17, or P35 (Figure 4).

**Figure 5:**
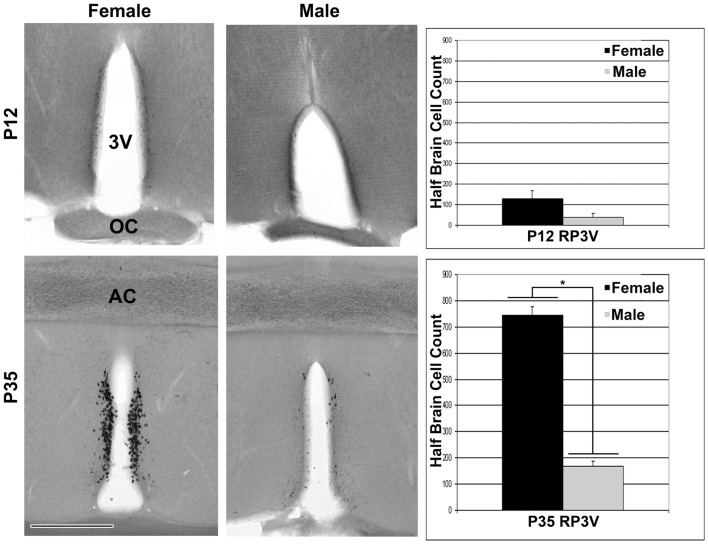
**Expression of Kiss1 in the RP3V**. Digital images (left) of coronal sections show that *Kiss1* in the RP3V was first detected at P12. A dramatic sex difference in the number of cells expressing *Kiss1* was evident around the time of puberty onset in females, P35 (see graphs on the right; *n* = 3 per group; mean ± SEM; *p < 0.01). 3V, third ventricle; OC, optic chiasm, AC, anterior commissure; Scale bar = 500 μm.

In rostral sections, *Kiss1* was first detected using ISH in a few cells at P12 (Figure 5). As with the more caudal ARC population, there was a distribution of cells in the rostral sections that begins near the opening of the third ventricle and extends caudally in a continuous cluster beyond the level of the anterior commissure. For the entire distribution, *Kiss1* was found in cell bodies along a narrow periventricular strip noted as the rostral periventricular region of the third ventricle (RP3V) (4). At P12 there was not a statistically significant sex difference in the number of cells found in the rostral population of cells. At P35 there appeared dramatically more cells in RP3V sections in both sexes as well as a significant sex difference in the number of cells found in these sections [Figure 5; *F*(1,4) = 262.0, *p* < 0.01], but no difference in the total number of cells in the ARC sections [Figure 5; *F*(1,4) = 1.83, *p* = 0.25].

### Sex differences in absolute *Kiss1* amount in fetal and adult mouse brain

**Figure 6:**
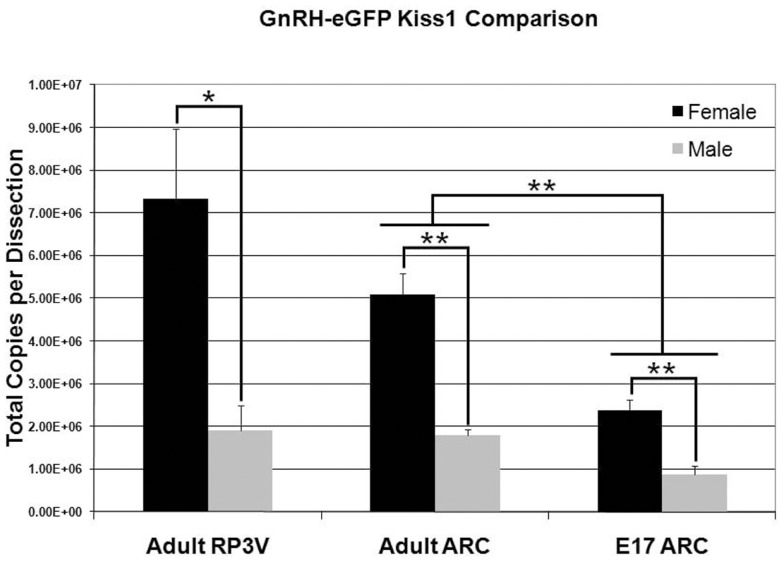
**Sex and age differences in *Kiss1* expression**. The visual sex difference in the amount of *Kiss1* detected by ISH in hypothalamic tissue was confirmed by QPCR. There was a significant difference in total copy number between males and females at both ages in the ARC and in the RP3V of adults. In addition, a *post hoc* analysis showed a significant difference in the amount of *Kiss1* between adults and embryos in the ARC (*n* = 3 for each group; data is mean ± SEM; *p < 0.05, **p < 0.01).

Quantitative reverse transcription real-time PCR was used to assess the absolute level of *Kiss1* in E17 and P50 (young adult) male and female GnRH-eGFP mice. As previous results suggested, and in agreement with the current ISH data, adult females expressed nearly four times as much *Kiss1* as males in the RP3V [Figure 6; *F*(1,4) = 9.77; *p* < 0.05]. In the caudal hypothalamic region of the adult animals, females were found to express more than twice as much transcript as males [Figure 6; *F*(1,4) = 43.1, *p* < 0.01]. A similar difference was found in the E17 animals, when *Kiss1* expression is restricted to the caudal region around the ARC, with females expressing more than twice as much *Kiss1* as males [Figure 6; *F*(1,6) = 22.1; *p* < 0.01]. Females were not examined at the time of dissection to determine estrous stage. In a final comparison, and in agreement with the increase in cell count with age, the level of *Kiss1* expression was significantly different between fetal and adult animals [Figure 6; age effect: *F*(1,10) = 41.01, *p* < 0.01; sex by age effect; *F*(1,10) = 10.26, *p* < 0.01].

### Potential source of sex differences in *Kiss1* in fetal mouse brain

**Figure 7:**
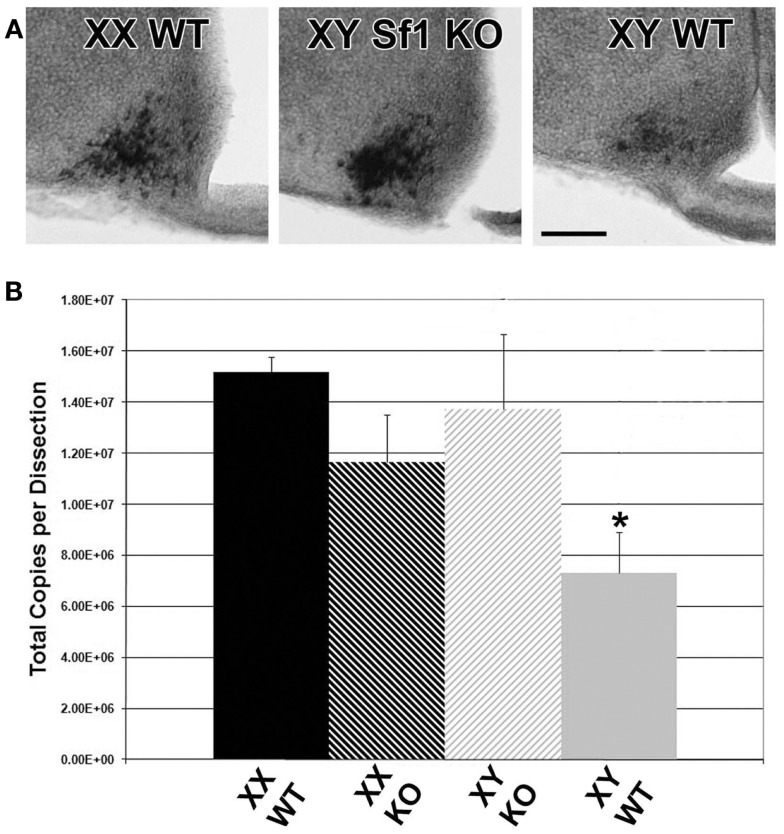
**Pre-natal *Kiss1* expression is gonad dependent**. Digital images of coronal sections in **(A)** show that E17 *Sf1* KO XY resemble WT XX more than WT XY mice by qualitative inspection of ISH reaction product. QPCR quantification of *Kiss1* **(B)** reveals a significant effect of sex by genotype with WT males expressing less *Kiss1* than KO males or females of either genotype (*n* = 3 per group; mean ± SEM. After a significant interaction between sex and genotype, *p < 0.05 indicates a specific of XY KO to XX WT. 3V, third ventricle; ME, median eminence. Scale bar = 100 μm.

To determine if the sex difference of *Kiss1* in ARC neurons was related to steroid hormones or gonadal status of fetal mice, we conducted *Kiss1* ISH analyses of *Sf1* KO XY mice (agonadal due to disruption of the *Sf1* gene but genetically male) compared with XY and XX WT littermates. *Sf1* KO mice die shortly after birth due to the absence of adrenal hormones, and thus without glucocorticoid supplementation these animals can only be evaluated at embryonic ages (24, 25). However, the advantage of using these animals is that the effects of total gonadal ablation can be achieved from the earliest stage of development, before surgical gonadectomy is possible. In each experiment, sections from *Sf1* KO XY mice were most comparable to sections from WT XX mice by qualitative visual inspection of the optical density of reaction product (Figure 7). Cells observed in sections from WT XY mice were consistently lighter in color than either WT XX or XY *Sf1* KO mice.

To verify that the visual ISH difference represents an actual difference in *Kiss1*, QPCR was performed on cDNA from hypothalamic tissue dissections from E17 XY and XX *Sf1* KO and WT littermates. There was a sex difference in *Kiss1* copy number between XY and XX WT mice from the *Sf1* KO line (*p* < 0.05 *post hoc*) that was similar to the results from the previous QPCR experiments using GnRH-eGFP animals. Importantly, there was a significant sex by genotype interaction [Figure 7; *F*(1,8) = 6.56; *p* = 0.034] with XY WT mice expressing significantly less *Kiss1* than XY KO, but with no difference between XX WT and XX KO. Thus the impact of the KO was only seen in males that may have had functional gonads.

## Discussion

An increasing number of studies have indicated the importance of kisspeptin signaling for both the onset of puberty and the promotion of normal adult reproduction (1, 26). Several have demonstrated the sex-steroid dependence of peri-pubertal changes in Kiss1 expression and sexually dimorphic Kiss1 expression in the rostral hypothalamus (11, 27). However, despite the pre-pubertal sex differences in circulating sex steroids, most notably at certain perinatal “critical periods,” until recently few studies have investigated the early developmental expression of Kiss1 and its cognate receptor KISS1R (12, 28).

In the last few years a growing body of research has begun to shed light on the emerging understanding that the kisspeptin system is actively regulated long before puberty. Several recent reports have examined the expression of *Kiss1* early in life. In a study of the ontogeny of kisspeptin neurons using BrdU birth dating in rats, these neurons differentiate locally within the region of the ARC beginning around E12.5 (29). *Kiss1* was present in hypothalamic micropunches and KISS1 immunoreactive cells and fibers were detected as early as E14.5. Importantly, by showing that the number of cells increased early in development (E14.5–18.5) and then decreased (E18.5–22.5) the data suggests pre-natal regulation of the kisspeptin system. Similar to these results, *Kiss1* expressing neurons were detected via ISH soon after birth (first reported for P3) in the ARC of rats and there were transient sex differences in the number of *Kiss1* cells, with females having significantly more cells at certain times than their male littermates (30). Finally, in mice examined for *Kiss1* expression on the day of birth, both the number of *Kiss1* positive cells and the amount of *Kiss1* per cell were higher in females than males (31). Interestingly, by examining the levels of circulating testosterone and using transgenic models with disrupted kisspeptin or GnRH signaling, the authors demonstrated that sex differences in gonadal steroid production arise independent of KISS1/KISS1R and GnRH.

In the current series of experiments, *Kiss1* and *Kiss1r* were detected in E13 mouse brain tissue. Using IHC it was further demonstrated that *Kiss1* is translated into immunoreactive peptide as early as E17 and potentially earlier. This result is similar to findings of pre-natal kisspeptin expression in rats (29). The differences with our results, most notably that a small number of KISS1 IR cells and fibers were found in the RP3V pre-natally in rats, are likely due to species variation or differences in detection technique. The current results replicate earlier studies showing the sexually dimorphic number of *Kiss1* expressing cells in the ARC and RP3V of mice and rats (5, 31, 32), but importantly show that there is already a sexually dimorphic pattern within the fetal ARC, whether examined qualitatively by visual inspection of colorimetric ISH (see Figure 7) or quantitatively using QPCR. Our data show sex differences in the amount of ARC *Kiss1* at all ages examined, with RP3V expression only detected after birth. By using *Sf1* KO mice that do not develop gonads, the early dependence of *Kiss1* levels on gonadal status is strongly suggested. Although it is possible that knocking out *Sf1* causes changes in *Kiss1* expression in gonad independent manner – such as preventing signaling within the mediobasal hypothalamus itself – this is unlikely considering that XX knockouts (KO) were not significantly different from their WT littermates. The demonstration of regulated *Kiss1* expression early in development suggests functional relevance that merits further investigation.

In an effort to better understand the development of sex differences in the brain’s reproductive axis, the developmental profile of *Kiss1* expression using ISH was determined. Expression of *Kiss1* was found at E13 followed by a dramatic increase in the number of detectable cells positive for *Kiss1* by E15. This increase demonstrates an early expression of *Kiss1*, and also suggests a temporally coordinated up-regulation. From E15 to P0 there was no significant change in either the number of cells found to express *Kiss1* or in the distribution pattern of these cells around the ARC (i.e., ∼400 cells/half-brain distributed over ∼800 μm rostrocaudally). Protein translation from *Kiss1* was verified by IHC for KISS1 in E17 and adult animals where immunoreactive product was seen in a pattern similar to that seen with ISH. At all ages examined, mRNA coding for the kisspeptin receptor KISS1R also was detected. The presence of both the ligand and cognate receptor establishes the possibility that a functional signaling system exists not only prior to puberty, but also well before birth. KISS1R is highly selective for kisspeptin when compared with all other RF-amide peptides. Similar to previous results, at E13 these cells were found exclusively in the nasal compartment in a distribution pattern that strongly resembles the migrating GnRH neuron population (33). Co-localization of immunoreactive GnRH and *Kiss1r* in E15 animals confirmed that cells seen to contain *Kiss1r* are, in fact, migrating GnRH neurons. Unfortunately, the absence of verified antibodies against KISS1R prevented the examination of the translation status of KISS1R.

Projections from neurons in the ARC may develop relatively late (34, 35). This would make ARC KISS1 an unlikely source of ligand for migrating GnRH neurons at embryonic ages, particularly when they are in the nasal compartment expressing *Kiss1r*. Previous results showing the efficacy of peripheral kisspeptin administration suggest that systemic, endocrine-like signaling, as opposed to synaptic release, could potentially play a role in development (36). Other peripheral sources of kisspeptin are possible (e.g., placenta), which would suggest that signaling is not for the purpose of providing a directional cue for migrating GnRH neurons but could possibly influence axon targeting (37). Similar to previous findings, one additional population of *Kiss1r* expressing cells was found in the habenula (38). These cells are located at the approximate border of the medial and lateral divisions of the habenula and were seen from E17 on (though not noted in younger animals). It is interesting to note the role of the habenula in physiological changes related to seasonality and specifically in seasonal breeders (36, 39, 40). While it remains to be determined where the ligand would come from to reach cells in the habenula expressing KISS1R, one possible source is the “estrogen concentrating cells” of the POA that send projections to the habenula (41–43). This also raises the possibility that patients with inactivating mutations of KISS1R or KISS1 may exhibit phenotypes other than those manifested in the reproductive system.

The data from older animals in the current study confirms the pattern of *Kiss1* expression that has been reported previously (5, 15, 44). Specifically, *Kiss1* was observed in the ARC in both sexes at all post-natal ages in a pattern very similar to the fetal pattern. At P12 there was a significant sex difference in the number of *Kiss1* expressing cells in the ARC, with females having more cells than males. Since the number of cells in males is similar to that of females by the next time point (P35) it could simply indicate a sex difference in maturational rate, which would not be unusual for the development of brain characteristics (45). Similarly, it is possible that all post-natal sex differences observed in the ARC are due to differences in circulating sex steroids; several studies have shown that sex differences in *Kiss1* expression in the RP3V are eliminated in mice and rats that have artificially equal levels of sex steroids (5, 14). Interestingly, P12 is the first age at which *Kiss1* expression was detected in a few cells in rostral sections. This result is similar to previously published research showing detection of *Kiss1* at P10 (32). At this age, possibly due to the apparently low level of message or the relatively low number of cells, there was no sex difference in the number of Kiss1 containing cells seen in the RP3V. By contrast, at around the time of puberty onset (P35) there was a dramatic sex difference in the number of *Kiss1* expressing cells in the RP3V, with females having far more cells than males.

In the current study, data from three different approaches suggest that the level of *Kiss1* in the ARC is a reliable sex difference in development and adulthood. First, qualitative differences were noted between males and females based on the optical density of ISH reaction product. Second, quantitative QPCR produced complementary results. The complementary nature of both ISH and QPCR assay was particularly evident at E17. Although the number of cells positive for Kiss1 in the ARC appeared similar according to ISH, females had absolute levels of *Kiss1* ∼twofold greater than males according to QPCR and this difference remained at P35. As a verification of the method, the sex difference in adult *Kiss1* levels within the more rostral RP3V was approximately fourfold as expected from data in reports for either whole hypothalamus or RP3V, respectively (28, 46). It should be noted that the adult females used for QRCR experiments were not examined to determine which stage of the estrous cycle they were in at the time of sacrifice, nor were circulating levels of sex steroids measured in either males or females. It has been established that *Kiss1* levels depend on levels of circulating sex steroids and varies dramatically during the estrous cycle in both RP3V and ARC (47). It is possible that the observed sex differences are due, fully or in part, to differences in circulating levels of sex steroids. Finally, as many sex differences in adulthood are the direct result of differences in the steroid environment of the fetus (48), agonadal *Sf1* KO mice were used to further test the gonadal steroid dependence of pre-natal *Kiss1* expression. The intensity of ISH reaction product revealed that *Sf1* KO XY mice were more similar to XX WT rather than XY WT mice, suggesting that the level of *Kiss1* expression is directly dependent on the gonadal status and presumably the pre-natal steroid environment.

Pre-natal sexual differentiation of the rodent brain is largely driven by testosterone produced by the developing testes that is converted to estradiol within the brain itself. Female fetuses are protected from masculinization and defeminization by circulating alpha-fetoprotein which binds circulating estrogens and prevents it from acting in the brain (49). Based on the finding that estradiol administration to adult animals decreases ARC *Kiss1* expression while increasing expression in the RP3V (5, 14), it is reasonable to speculate that testosterone derived estradiol inhibits *Kiss1* expression in male fetuses. This also implies that there is another mechanism in addition to estradiol stimulation that drives expression of kisspeptin in the RP3V as no rostral *Kiss1* expression was detected in any animals, regardless of genotype, prior to P12. If estradiol signaling were the only driving force for RP3V expression, it would be reasonable to expect expression in fetal males. Previous studies have shown that the RP3V kisspeptin expressing population can be decreased by estradiol administration at birth (50, 51) or increased by neonatal castration (46), as well as decreased by gonadectomy later during pre-pubertal development (27). This may indicate that the RP3V population becomes sensitized to estradiol later during development and that other developmental events are required prior to puberty.

In summary, *Kiss1* and *Kiss1r* were detected in fetal mice early in development. *Kiss1r* was co-localized with GnRH in the nasal compartment and rostral forebrain during GnRH neuron migration, while *Kiss1* was found selectively in cells within the developing ARC. At E17 KISS1 was observed in the same region and distribution as the mRNA. *Kiss1* expression was both qualitatively and quantitatively sexually dimorphic, and likely related to hormone exposure prior to birth. The sex difference in this region is maintained into adulthood, although the exact cause and function of this early kisspeptin expression remains to be elucidated.

## Conflict of Interest Statement

The authors declare that the research was conducted in the absence of any commercial or financial relationships that could be construed as a potential conflict of interest.
